# What is normal? Next generation sequencing-driven analysis of the human circulating miRNAOme

**DOI:** 10.1186/s12867-016-0057-9

**Published:** 2016-02-09

**Authors:** D. P. Tonge, T. W. Gant

**Affiliations:** School of Life Sciences, Faculty of Natural Sciences, Keele University, Keele, ST5 5BG UK; Centre for Radiation, Chemical and Environmental Hazards, Public Health England, Harwell Science and Innovation Campus, Didcot, OX11 0RQ UK

**Keywords:** Plasma sequencing, miRNA, Small RNA, NGS, Biomarker, Baseline

## Abstract

**Background:**

MicroRNAs (miRNAs) are short non-protein-coding RNA species that have a regulatory function in modulating protein translation and degradation of specific mRNAs. MicroRNAs are estimated to target approximately 60 % of all human mRNAs and are associated with the regulation of all physiological processes. Similar to many messenger RNAs (mRNA), miRNAs exhibit marked tissue specificity, and appear to be dysregulated in response to specific pathological conditions. Perhaps, one of the most significant findings is that miRNAs are detectable in various biological fluids and are stable during routine clinical processing, paving the way for their use as novel biomarkers. Despite an increasing number of publications reporting individual miRNAs or miRNA signatures to be diagnostic of disease or indicative of response to therapy, there is still a paucity of baseline data necessary for their validation. To this end, we utilised state of the art sequencing technologies to determine the global expression of all circulating miRNAs within the plasma of 18 disease-free human subjects.

**Results:**

In excess of 500 miRNAs were detected in our study population with expression levels across several orders of magnitude. Ten highly expressed miRNAs accounted for 90 % of the total reads that mapped showing that despite the range of miRNAs present, the total miRNA load of the plasma was predominated by just these few species (50 % of which are blood cell associated). Ranges of expression were determined for all miRNA detected (>500) and a set of highly stable miRNAs identified. Finally, the effects of gender, smoking status and body mass index on miRNA expression were determined.

**Conclusions:**

The data contained within will be of particular use to researchers performing miRNA-based biomarker screening in plasma and allow shortlisting of candidates a priori to expedite discovery or reduce costs as required.

**Electronic supplementary material:**

The online version of this article (doi:10.1186/s12867-016-0057-9) contains supplementary material, which is available to authorized users.

## Background

MicroRNAs (miRNAs) are short non-protein-coding RNA species that have a regulatory function in modulating protein translation from specific mRNAs [3]. MicroRNAs are estimated to target approximately 60 % of all human mRNAs [7] and are associated with the regulation of all physiological processes. Similar to many messenger RNAs (mRNA), miRNAs exhibit marked tissue specificity [9, 12], and appear to be dysregulated in response to specific pathological conditions [3]. Perhaps most significant is the finding that miRNAs are detectable in various biological fluids [19] and are stable during routine clinical processing [13], paving the way for their use as novel biomarkers. Whilst the presence of extra-cellular miRNAs in a range of biological fluids has been consistently described within the recent literature, the precise mechanisms via which they leave their “host cell” and enter the circulation are still under investigation. Nevertheless, it is now accepted that miRNAs are not simply shed from necrotic or apoptotic cells but rather, are subject to selective export from specific cells and function as extra-cellular signalling molecules, retaining their biological activity in recipient cells [1].

In considering the use of miRNAs as novel biomarkers, it is essential to generate baseline data that describes which miRNA species are present in a given biological fluid, where they likely originate from, and what is considered normal in terms of their patterns of expression. Indeed, we take such data for granted when we consider other routine clinical analyses such as the measurement of plasma/serum aspartate aminotransferase and alanine aminotransferase for the determination of liver function. Despite an increasing number of publications (5897 articles indexed by PubMed at the time of publication) reporting individual miRNAs or miRNA signatures as biomarkers of various conditions, there is still a paucity of baseline data necessary for their validation. To date, there are no comprehensive assessments of plasma miRNA expression conducted using unbiased methodology (i.e. where the miRNA targets are not required a priori) in a representative set of healthy human subjects reported. To this end, we utilised state of the art sequencing and bioinformatic techniques to determine the global expression of all circulating miRNAs within the plasma of 18 disease-free human subjects with high resolution. We report data to support the key questions outlined above including (1) a comprehensive list of all miRNAs found within human plasma, (2) the expected range of expression in a disease-free cohort and (3) the likely tissue of origin for a selection of the most highly expressed miRNAs. Furthermore, we report the effects of sex, smoking status and differing body mass index (BMI) on these parameters.

## Results and discussion

**Fig. 1:**
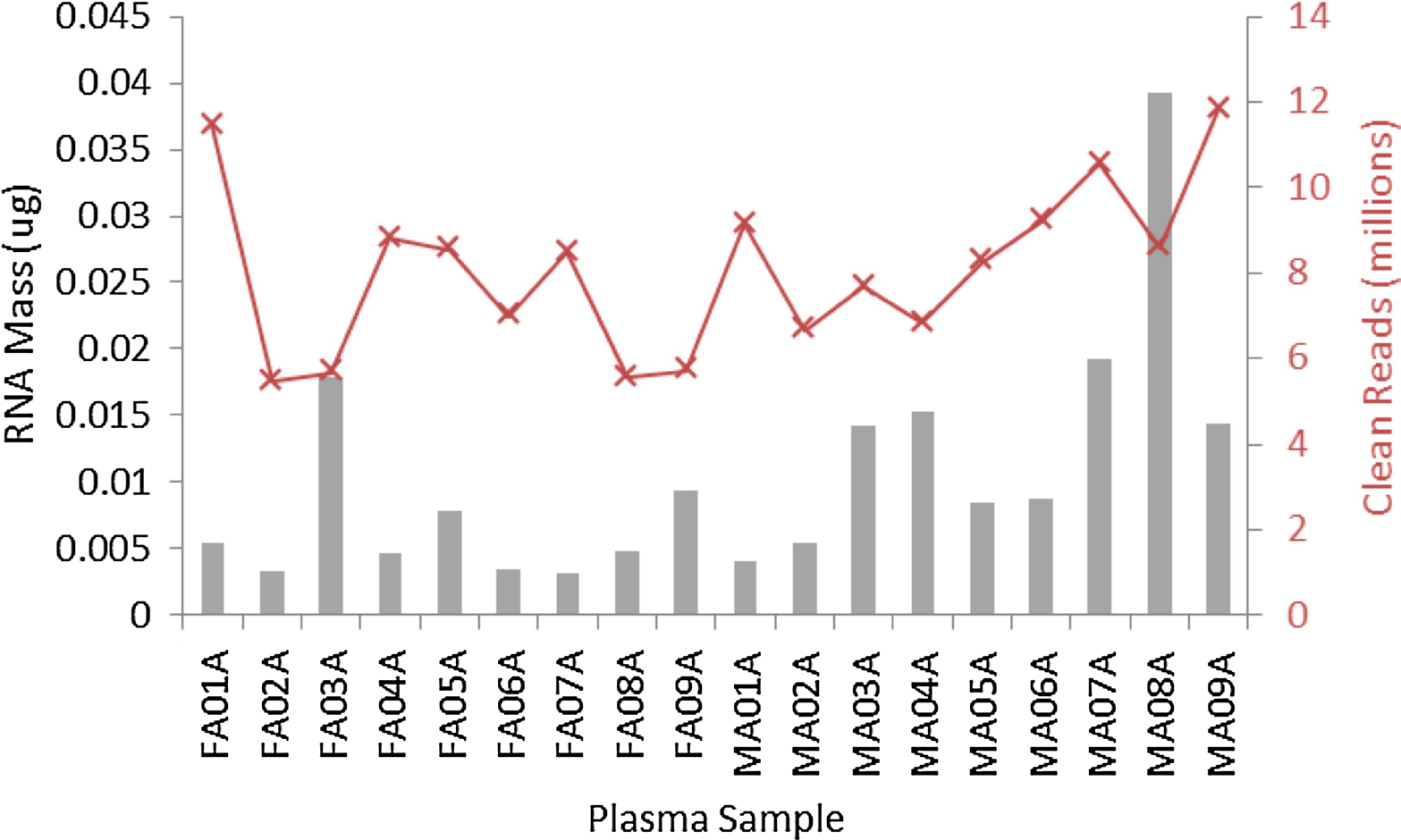
Mass of RNA obtained in micrograms from 5 mL plasma (*grey bars*) for each donor specimen against the number of clean sequencing reads obtained in millions (Q ≥ 30) (*red crosses*)

**Fig. 2:**
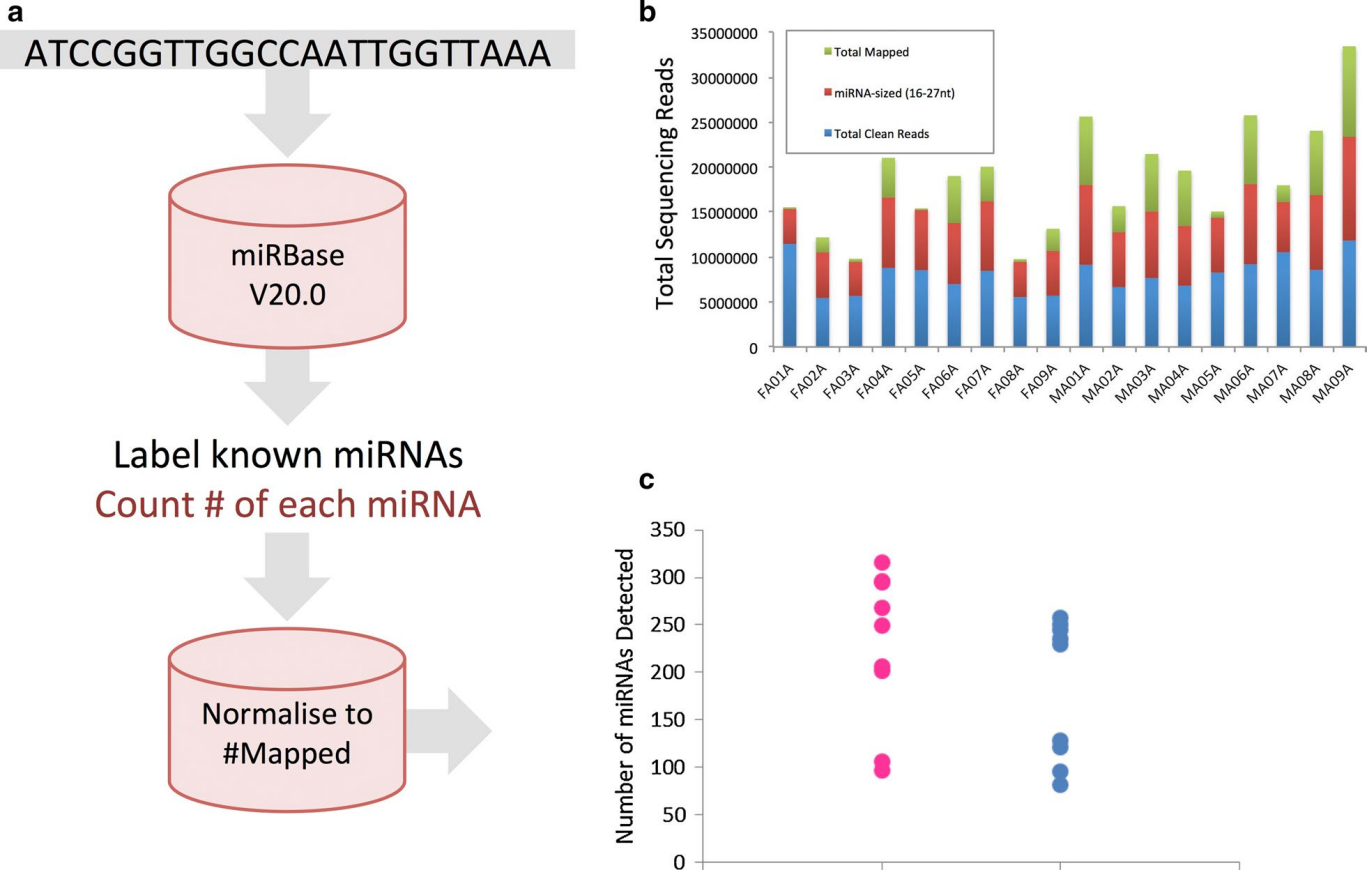
**a** Schematic showing the bioinformatic approach taken for miRNA sequencing and mapping. **b** Number of clean sequencing reads obtained in millions (Q ≥ 30) (sum of *blue*, *red* and *green bar* elements), total number of reads of miRNA size (sum of *red* and *green* elements) and the total number of reads mapped to miRBase (*green* element) for each study participant. **c** Total number of miRNAs mapped for each study participant sorted according to gender (*blue*—males, *pink*—females) (n = 9, N = 18). Note, only those miRNAs detected by at least ten independent sequencing reads, and that were present in at least three study participants are included

All data presented herein are derived from 18 human plasma specimens, details of which can be found in Table 1. Circulating RNA was extracted from 5 mL of each plasma sample, yielding an average RNA mass of 0.01 µg (Table 2). The mass of RNA obtained was consistent irrespective of gender (P > 0.05; two-tailed t test; n = 9). Next generation sequencing for small RNA molecules yielded an average of 8,080,000 high quality (≥Q30) sequencing reads per sample (Fig. 1). Raw sequencing data in fastq format can be obtained from the Short Read Archive (SRA), accession number SRP064104. Mapping of the raw sequencing data to miRBase version 20.0 (Fig. 2a) identified 548 different miRNAs across the 18 samples analysed. Of these, only 53 were present in all samples (Table 3). The number of reads mapped and the number of miRNAs identified in each plasma sample is detailed in Fig. 2b, c respectively.

**Table 1:** Details of the donor population

Sample number	Sample name	Sex	Age (range)	Smoking	BMI (range)	Obese
1	FA01	F	40 (20–61)	(4/9)	35 (24–46)	(7/9)
2	FA02					
3	FA03					
4	FA04					
5	FA05					
6	FA06					
7	FA07					
8	FA08					
9	FA09					
10	MA01	M	39 (19–67)	(4/9)	28 (18–47)	(2/9)
11	MA02					
12	MA03					
13	MA04					
14	MA05					
15	MA06					
16	MA07					
17	MA08					
18	MA09					

Age—rounded to the nearest whole year, Smoking status—self reported, *BMI* body mass index (patients with a BMI > 30 were considered obese)

**Table 2:** Total RNA concentration derived from 5 mL of human plasma

Sample number	Sample name	Total mass RNA (µg)
1	FA01A	0.0054
2	FA02A	0.0033
3	FA03A	0.0179
4	FA04A	0.0046
5	FA05A	0.0079
6	FA06A	0.0034
7	FA07A	0.0032
8	FA08A	0.0048
9	FA09A	0.0093
10	MA01A	0.0041
11	MA02A	0.0054
12	MA03A	0.0143
13	MA04A	0.0153
14	MA05A	0.0084
15	MA06A	0.0088
16	MA07A	0.0193
17	MA08A	0.0392
18	MA09A	0.0144

Samples prefixed with FA are from female donors, those prefixed with MA are from male donors. RNA concentrations determined using the QuBit fluorimeter, invitrogen

**Table 3:** Details of miRNAs detected in all plasma samples analysed (18 out of 18 individuals)

miRNA	Normalised expression
hsa-miR-486-5p	56.91769
hsa-miR-25-3p	0.263462
hsa-miR-10b-5p	14.94519
hsa-miR-99b-5p	0.033431
hsa-let-7f-5p	0.858338
hsa-miR-10a-5p	1.412167
hsa-miR-423-3p	0.060593
hsa-miR-101-3p	0.046649
hsa-miR-103a-3p	0.037811
hsa-miR-191-5p	0.548739
hsa-miR-423-5p	2.303739
hsa-miR-182-5p	0.201054
hsa-miR-30a-5p	0.134701
hsa-let-7a-5p	0.515206
hsa-miR-21-5p	0.215671
hsa-miR-150-5p	0.235879
hsa-miR-22-3p	1.557981
hsa-miR-28-3p	0.305134
hsa-let-7i-5p	0.186836
hsa-miR-30e-5p	0.036269
hsa-miR-16-5p	0.07779
hsa-miR-92a-3p	2.20284
hsa-miR-126-5p	0.271752
hsa-miR-30d-5p	0.105128
hsa-miR-451a	0.041124
hsa-miR-92b-3p	0.129143
hsa-miR-26a-5p	0.211624
hsa-miR-140-3p	0.034428
hsa-miR-375	0.295206
hsa-miR-27b-3p	0.273707
hsa-miR-143-3p	0.423736
hsa-miR-328	0.021033
hsa-miR-146b-5p	0.094907
hsa-miR-125a-5p	0.062047
hsa-miR-1246	0.050083
hsa-miR-181a-5p	1.238587
hsa-miR-222-3p	0.026529
hsa-miR-192-5p	0.196152
hsa-miR-148a-3p	0.102086
hsa-miR-146a-5p	0.399876
hsa-let-7b-5p	0.051522
hsa-miR-127-3p	0.094643
hsa-miR-151a-3p	1.164533
hsa-miR-409-3p	0.141178
hsa-miR-584-5p	0.058182
hsa-miR-186-5p	0.095506
hsa-miR-378a-3p	0.559047
hsa-miR-320a	2.876669
hsa-miR-769-5p	0.113601
hsa-miR-181b-5p	0.09218
hsa-miR-184	0.083304
hsa-miR-4448	0.04067
hsa-miR-320d	0.035856

**Fig. 3:**
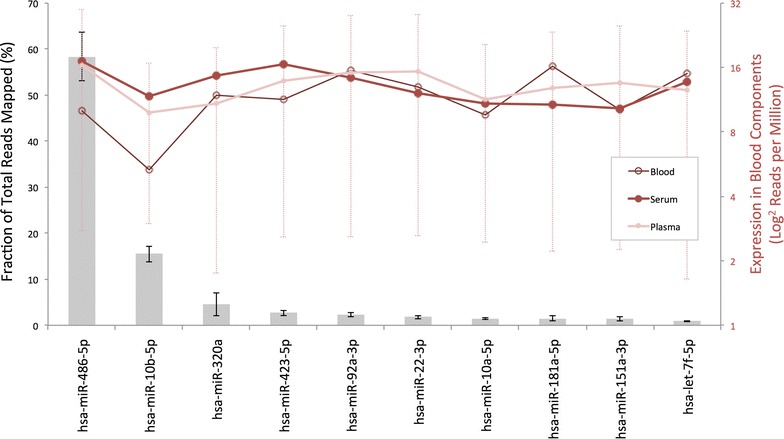
Fraction of the total sequencing reads mapped occupied by each of the ten most highly expressed miRNAs (based upon the mean of 18 samples) are presented (*grey bars*) ± S.E.M. The mean expression in human plasma, serum and whole blood derived from 116 publically available datasets is included on the* right hand axis* for comparison (reads per million—RPM ± S.E.M, in Log2 form). Note for clarity,* errors bars* are only included for the plasma datasets

Normalised miRNA expression values were used to compare the expression of individual miRNAs between plasma samples (Additional file 1: S1). In the first instance, we investigated which miRNAs were most abundantly expressed in human plasma. Based upon the mean expression of 18 independent samples, microRNAs 486-5p, 10b-5p, 320a, 423-5p, 92a-3p, 22-3p, 10a-5p, 181a-5p, 151a-3p, and let-7f-5p were found to be most abundantly expressed in the plasma of our study participants. Surprisingly, the most abundant miRNA (miR-486-5p) accounted for almost 60 % of the total number of sequencing reads mapping to miRNAs and furthermore, the ten most abundant miRNAs accounted for approximately 90 % of mapped reads. To verify these striking findings, we reviewed the results of thirty-seven publically available plasma miRNA-seq datasets obtained via miRmine online (Guan Laboratory, University of Michigan—Additional file 1: S1, sheet 2) and undertook a complete re-analysis (from raw sequencing data) of three randomly selected plasma sequencing runs from the short read archive [accession numbers; SRR1005875, SRR1005877 and SRR1005876]. The top ten miRNAs accounted for 68 and 74 % of the total mapped read count in the reviewed and re-analysed datasets respectively. The fraction of the total plasma miRNA read count attributed to each of the top 10 most highly expressed miRNAs is detailed in Fig. 3 (left-hand axis). The normalised expression (reads per million; RPM expressed in Log 2 scale) of each of these miRNAs in human whole blood, serum and plasma (using publically available data via miRmine) is included on the right-hand axis. Comparison of plasma data from this study and those derived from miRMine revealed no clear correlation, however, significant variation within the later dataset may explain this finding (Fig. 3, right-hand axis error bars).

**Fig. 4:**
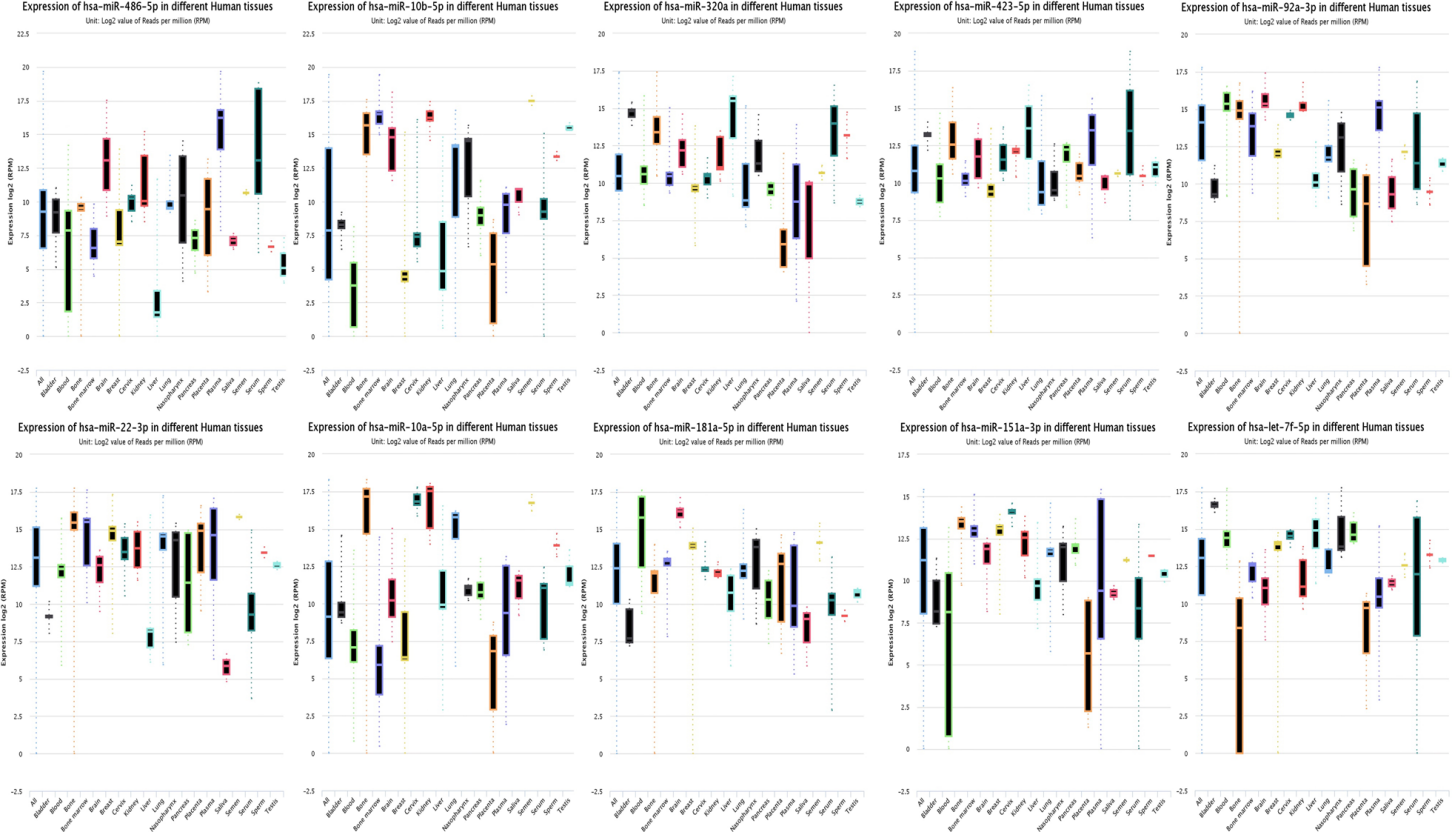
Tissue based expression data for the ten most highly expressed miRNAs detected in human plasma. Data are reads per million (RPM) in Log 2 form obtained from miRMine online (Guan Laboratory, University of Michigan). *Box* and *whisker plots* identify the minimum and maximum, upper and lower quartile and median values

Next we sought to determine the origin of these highly expressed miRNAs. As per the available literature, five out of the ten most highly expressed miRNAs were previously identified as being highly expressed by cells of the blood. MicroRNAs 486-5p and 92a-3p were shown to be highly expressed by erythrocytes, whilst miRNAs 181a-3p, 151a-3p and let-7f-5p were highly expressed by various blood cell types [11, 15]. To ensure our highly expressed miRNAs were naturally present at such levels, as opposed to the result of haemolysis, we determined the expression variation in both blood cell and non-blood cell associated miRNAs with the expectation that these should be equivalent in the absence of haemolysis. Variation in our blood cell associated miRNAs was in fact less than that of the non-blood cell associated miRNAs (Coefficient of variation = 95.8 vs 116.3 %, N = 18), supporting our findings. Leveraging over 300 publically available next generation sequencing datasets (including 66, 13 and 37 in human blood, serum and plasma respectively), we were able to determine the tissue expression profile of each highly expressed miRNA. Whilst each miRNA exhibited a different tissue based expression pattern, nine of the ten (miRNA-151-3p was the exception) highly expressed miRNAs identified herein had median expression levels in either blood, serum or plasma in excess of the median expression for all tissues, suggesting that the blood is a significant source of these. Furthermore, in considering only those miRNA-seq datasets generated from human plasma, nine of the ten (miR-10b-5p was the exception) top expressed miRNAs identified in this study appeared within the upper quartile of expression, confirming that our results mirror those of other studies conducted using similar methods in the same biological sample type. Comprehensive tissue expression data for the most highly expressed miRNAs identified in human plasma is included within Fig. 4.

Our realisation that a number of the most highly expressed miRNAs were of blood cell origin led us to consider the potential implications that this may have for biomarker development (for a comprehensive review of the challenges associated with miRNA biomarker development, please refer to [16]. The routine clinical sampling of blood involves venapuncture, delivery to a suitable storage vessel with or without anticoagulant and delivery to the analytical laboratory. The preparation of plasma or serum involves a further step during which blood cells are removed by centrifugation to leave a “cell free” supernatant. At each point, there is potential to influence the number of blood cell associated miRNAs present in the remaining sample either through haemolysis following venapuncture or during transportation, or via variation in the time and or intensity of centrifugation. In support of this, Pritchard and colleagues have previously demonstrated significant increases in erythrocyte-associated miRNAs (miRs-451, 16, 92a, 486) in haemolysis, and explained miR-150 and 223 expression as a function of lymphocyte and neutrophil count respectively [15]. Thus, small variations in blood sample preparation may lead to individual samples being enriched or depleted in certain blood cell types and thus have significant impacts upon the expression of specific miRNAs. Of concern is the fact that all of the blood cell associated miRNAs identified herein have been identified in biomarker screens and or proposed as novel biomarkers for various conditions (miR-486-5p—gastric adenocarcinoma, miR-92a-3p—colorectal cancer, miR-181-5p—endometrial carcinoma, miR-151a-3p—paracetamol toxicity, let-7f-5p—Alzheimer’s disease) and may thus be subject to the aforementioned issues.

**Fig. 5:**
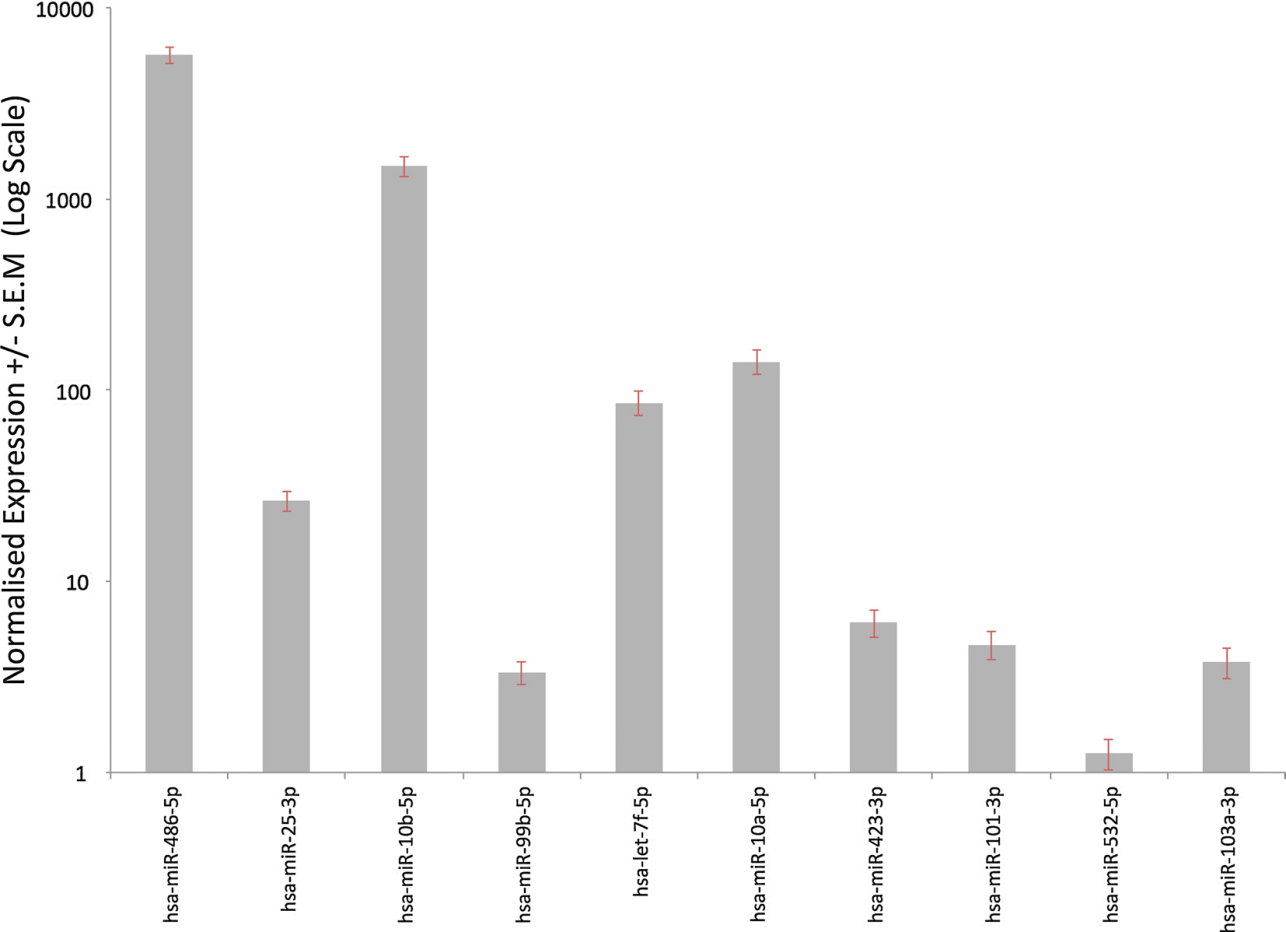
Mean normalized expression +/− standard error of the mean (S.E.M) for the ten most stably expressed miRNAs (N = 18)

In order to determine the normal range of circulating miRNA expression within our disease free population, the mean and the standard error of the normalised expression values was calculated and the miRNAs ranked according to their apparent stability. The most stably expressed miRNAs included microRNAs 486-5p, 25-3p, 10b-5p, 99b-5p, let-7f-5p, 10a-5p, 423-3p, 101-3p, 532-5p, and 103a-3p (Fig. 5). Although several of the most abundantly expressed miRNAs were identified, these highly stable miRNAs represented several orders of expression magnitude including both highly abundant and very lowly expressed miRNAs. Wang et al. [18] have previously reported the stability of a restricted set of plasma and serum miRNAs and despite differences in experimental design and measurement techniques, various miRNAs were identified as highly stable in both studies (Table 4).

**Table 4:** Comparison of the most stable miRNAs (90th percentile for stability) identified in this study with plasma and serum data from [18]

Wang et al. (top 10 % stable plasma miRNAs)	Wang et al. (top 10 % stable serum miRNAs)	Tonge et al. (top 10 % stable plasma miRNAs)
hsa-miR-106b	hsa-miR-106b	hsa-let-7a-5p
hsa-miR-16	hsa-miR-126	hsa-let-7f-5p
hsa-miR-185	hsa-miR-140-3p	hsa-let-7i-5p
hsa-miR-18a	hsa-miR-185	hsa-miR-101-3p
hsa-miR-19b	hsa-miR-191	hsa-miR-103a-3p
hsa-miR-222	hsa-miR-1974	*hsa-miR-106b-3p*
hsa-miR-320a	hsa-miR-1979	hsa-miR-10a-5p
hsa-miR-320b	hsa-miR-19b	hsa-miR-10b-5p
hsa-miR-324-3p	hsa-miR-320a	*hsa* - *miR* - *126* - *3p*
hsa-miR-345	hsa-miR-320b	*hsa* - *miR* - *126* - *5* ***p***
hsa-miR-346	hsa-miR-324-3p	hsa-miR-130a-3p
hsa-miR-484	hsa-miR-345	hsa-miR-130b-3p
hsa-miR-486-5p	hsa-miR-346	hsa-miR-148b-3p
hsa-miR-720	hsa-miR-451	hsa-miR-150-5p
hsa-miR-92a	hsa-miR-720	hsa-miR-151a-5p
	hsa-miR-92a	*hsa-miR-16-5p*
	hsa-miR-934	hsa-miR-181a-3p
		hsa-miR-182-5p
		*hsa* - *miR* - *191* - *5p*
		hsa-miR-21-5p
		hsa-miR-22-3p
		hsa-miR-221-3p
		hsa-miR-25-3p
		hsa-miR-26b-5p
		hsa-miR-28-3p
		hsa-miR-30a-3p
		hsa-miR-30a-5p
		hsa-miR-30d-5p
		hsa-miR-30e-5p
		hsa-miR-340-5p
		hsa-miR-342-3p
		hsa-miR-423-3p
		hsa-miR-423-5p
		*hsa* - *miR* - *451a*
		*hsa-miR-484*
		*hsa-miR-486-5p*
		hsa-miR-532-5p
		*hsa-miR-92a-3p*
		hsa-miR-92b-3p
		hsa-miR-99b-5p

Italicised miRNAs—identified as stable in plasma in both studies. Italicised and underlined miRNAs—identified as being stable in plasma herein, and in serum by Wang et al

**Fig. 6:**
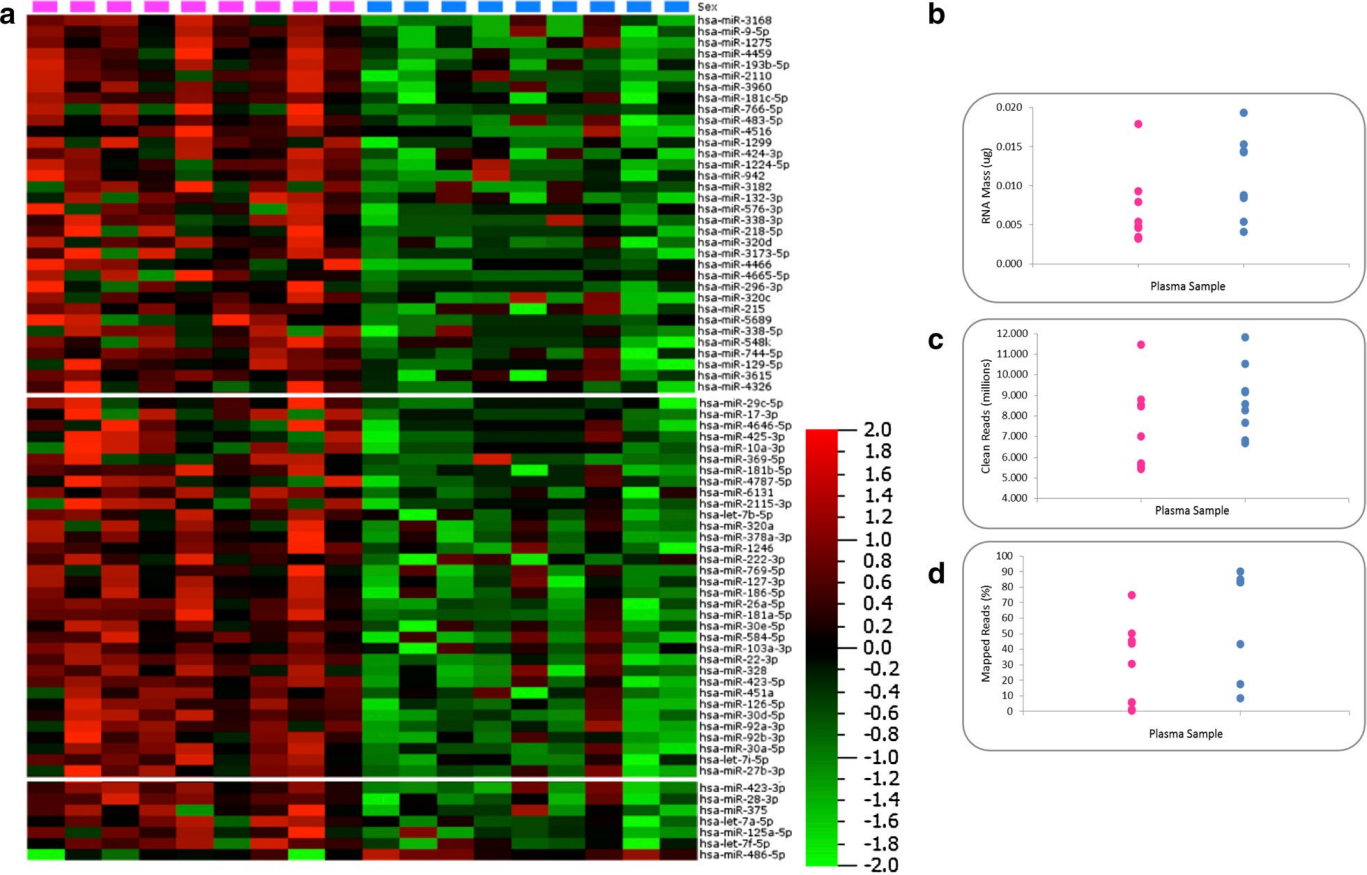
**a** Heat map diagram showing the effects of gender (*pink squares*—female, *blue squares*—male) on plasma miRNA expression. Data are normalised expression values determined by next generation sequencing, ordered by descending fold change where P < 0.05. The *colour bar* indicates the range of normalised expression values from −2 (*green*) to +2 (*red*). **b**–**d** RNA mass, number of clean sequencing reads and the proportion of mapped reads (%) separated by gender

The sole inclusion criterion for this study was the requirement to be free from overt disease at the time of blood donation and thus our patient cohort was diverse in their gender, smoking status and BMI. It was therefore possible to determine the effects of these factors on circulating miRNA expression. Perhaps unsurprisingly, the gender of the study participant had a significant effect on their circulating miRNA profile. Seventy-five miRNAs were differentially regulated between the two genders (Fig. 6a) however rather surprisingly 74 of these were up-regulated in females (in the absence of any significant differences in RNA mass obtained, number of clean sequencing reads or indeed the percentage of reads mapped to miRNAs—Fig. 6b, c). MicroRNA 486-5p, present in erythrocytes, was the only miRNA to be expressed at a higher level in males than in females, and we consider that this is likely due to the higher initial erythrocyte count that males present with. In considering why all of the deregulated miRNAs were elevated in the female sex, we determined the genomic location at which they were encoded with a hypothesis that there would be an over representation of miRNAs encoded by the X chromosome. In fact of the 75 miRNAs deregulated, only three were encoded at a locus on the X chromosome confirming that X copy number is unlikely responsible for this phenomenon. A review of the available literature reveals that few studies have previously investigated gender-specific miRNA expression, and there is an absence of those that have specifically considered gender-specific expression in healthy human subjects. In order to validate our findings, we therefore returned to the 37 publically available plasma miRNA-seq datasets, 27 of which had gender information. Considering the 74 miRNAs differentially expressed between male and female subjects in our study, we confirmed that 53 of these were also more highly expressed in the female sex of the publically available datasets.

**Fig. 7:**
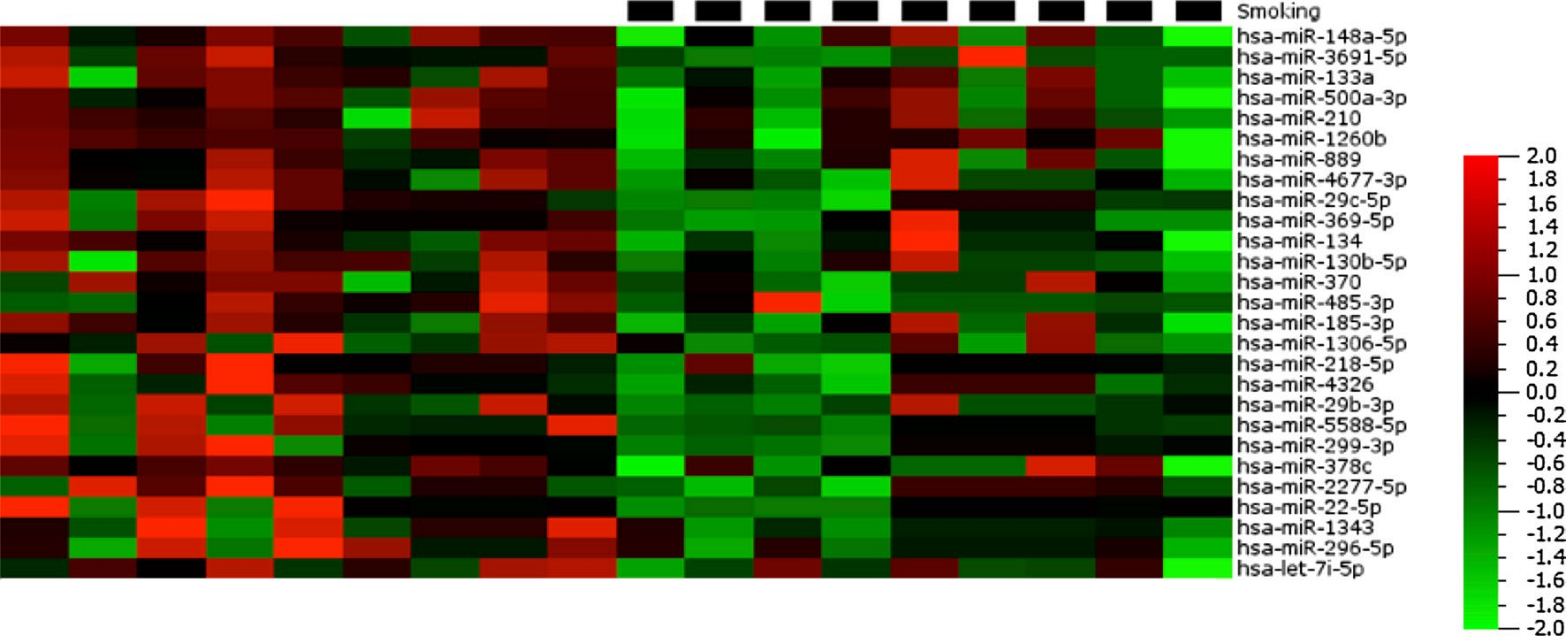
Heat map diagram showing the effects of smoking (*black squares*) on plasma miRNA expression. Data are normalised expression values determined by next generation sequencing, ordered by descending fold change where P < 0.05. The *colour bar* indicates the range of normalised expression values from −2 (*green*) to +2 (*red*)

We next considered the impact of smoking status on circulating miRNA expression. Of the 18 participants, 8 (45 %) were self-reported smokers (an equal number of males and females). Smoking was associated with the down-regulation of 27 plasma miRNAs (Fig. 7), including several previously identified as performing tumour suppressor-like functions; let-7i-5p [2], miR-148a-5p [22], miR-218-5p [17], miR-29-3p [20], miR-133a [4], miR-296-5p [10] and miR-370 [21]. We consider this finding particularly interesting; however, these results should be confirmed by analysis of a larger cohort of smokers incorporating robust statistical techniques to control for false discovery. Various publications have investigated the impact of smoking on miRNA expression in the lungs [8] and lung cells [5] and have reported an apparent global down-regulation of miRNA regulation, potentially mediated via disruption of the Erk/dicer/trbp pathway [14]. Here, we show the same phenomenon in the plasma however, there is little agreement at present between the dysregulated miRNAs identified in each individual study—perhaps reflecting a difference in the smoking cohort, experimental setup or suggesting that there are tissue specific effects.

**Fig. 8:**
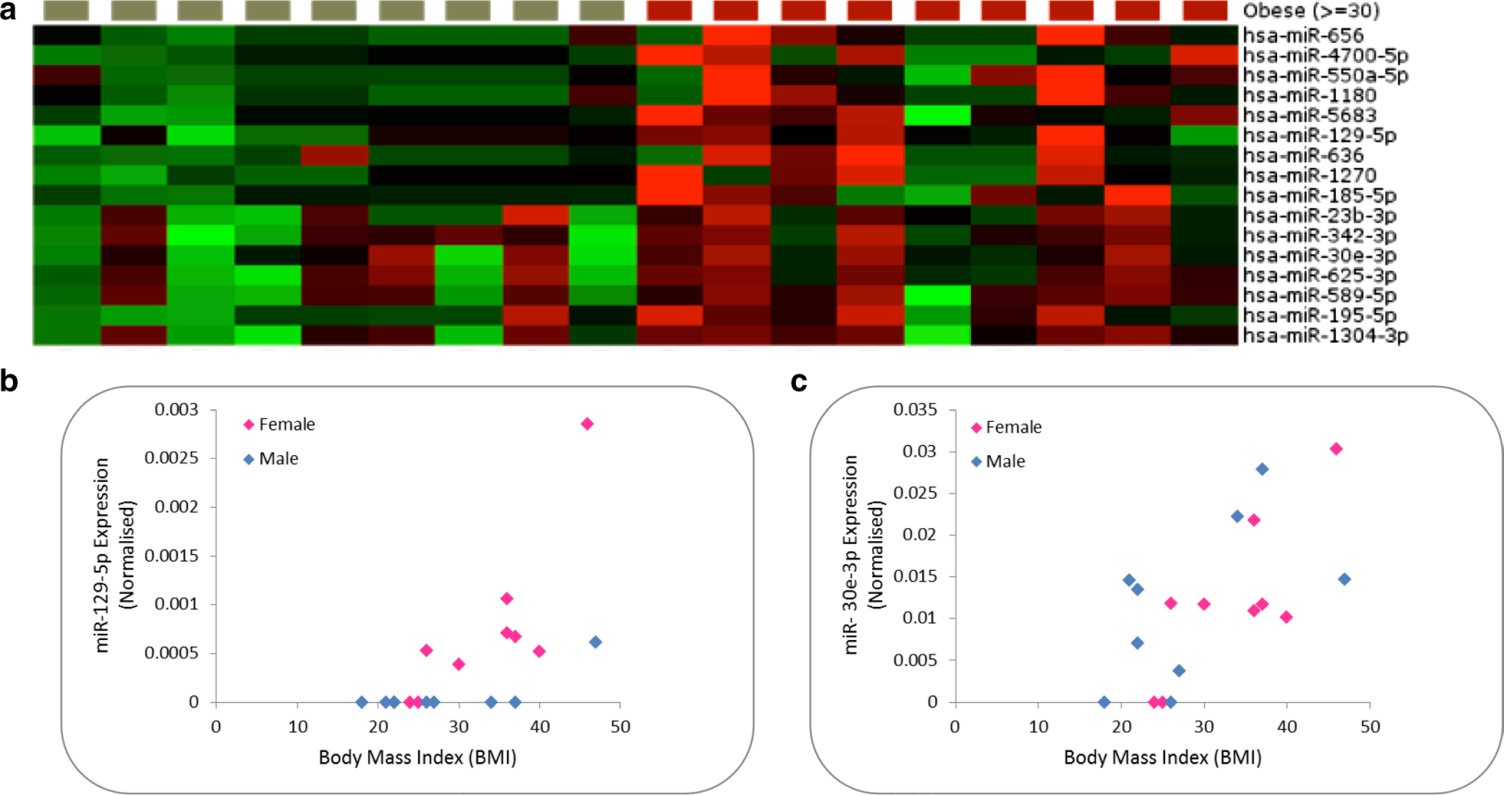
**a** Heat map diagram showing the effects of obesity (*red squares*) on plasma miRNA expression. Data are normalised expression values determined by next generation sequencing, ordered by descending fold change where P < 0.05. The *colour bar* indicates the range of normalised expression values from −2 (*green*) to +2 (*red*). **b**, **c** Correlation observed between normalised miR-129-5p and 30e-3p expression and body mass index (BMI) respectively

Finally, we considered the effects of obesity on the circulating miRNA profile of our study participants. Of the 18 donors, 8 had a BMI of greater or equal to 30 and were considered obese (2 males and 6 females). Sixteen miRNAs were dysregulated in obese individuals, and were more highly expressed compared to normal weight control subjects in all cases (Fig. 8a). Given that our donors had a range of BMI values, we sought to determine whether any of the obesity-associated miRNAs correlated with BMI. The expression of both miR-129-5p and miR-30e-3p were found to correlate with obesity (R = 0.67 and 0.64 respectively) (Fig. 8b, c). In both cases, the relationship was stronger amongst female participants however it is highly likely that this effect is down to a higher number of females than males being obese.

## Conclusions

The potential of miRNAs to serve as novel biomarkers is under intense investigation. To date, numerous studies have attempted to correlate miRNA expression with a range of endpoints. Despite the number of studies reporting such miRNA biomarkers, there is still a relative paucity of baseline data reporting the miRNA species expressed in a given biological fluid, what is considered normal in terms of their patterns of expression, and further, how this expression is altered by gender, obesity and smoking status. In this study we utilised state of the art sequencing technology to provide data to support the above key questions. In excess of 500 miRNAs were detected in our study population with expression across several orders of magnitude. However, only 53 of those miRNAs were detected in all participants with some miRNAs being detected in just a single individual. In considering relative expression between miRNAs, the top 10 most highly expressed candidates accounted for 90 % of the total reads that mapped to all miRNAs suggesting that despite the range of miRNAs present, the total miRNA load of the plasma was predominated by just 10 different species. Furthermore, many of the most abundant miRNAs have been shown to be highly expressed in cells of the blood and thus, perhaps with the exception of haematological biomarkers, their use as biomarkers should be approached with caution. Ranges of expression were determined for all miRNA detected (> 500) and a set of highly stable miRNAs identified. Finally, the effects of gender, smoking status and BMI on miRNA expression were determined. These data provide researchers with the ability to (1) determine the presence or absence of individual miRNAs within the plasma of a disease-free population, (2) consider the penetrance of each individual miRNA, (3) gain insight into the relative expression levels and “normal range” of individual miRNAs, ensuring that only suitabily highly expressed and stable candidates are taken forward, and (4) to have an indication of whether individual miRNAs are regulated by gender, smoking or obesity. Perhaps most striking and of most relevance here is the finding that of the large pool of miRNA detected in disease-free plasma, only a very small proportion of this may house suitable biomarker candidates (considering that, with the exception of the 10 most highly expressed miRNAs, only 10 % of the total miRNA pool is left and once miRNAs with low penetrance, those associated with cells of the blood, and those with high levels of inter-individual variation are discounted). Thus, the information contained within will assist researchers in designing more targeted miRNA biomarker studies.

## Methods

### Ethical approval and consent to participate

Sample collection was undertaken by Sera Lab ltd. who obtained ethical approval by an Institutional Review Board (Schulman Associates IRB #201209850). Written informed consent to participate was obtained from each study participant prior to sample collection.

### Consent for publication

The supplying company (Sera Lab ltd) obtained written consent to produce reports or articles about the study from each participant, subject to their names being omitted from any such publications.

### Samples, RNA isolation and sequencing

This study utilised human control material purchased from SeraLab ltd. All data were analysed anonymously; donor details are included in Table 1. Whole blood was drawn into EDTA containing tubes and stored on ice prior to centrifugation at 1000×*g* to obtain the plasma component. Plasma samples were frozen at −20 °C immediately after production. RNA was extracted from 5 mL of each plasma sample using the QiaAmp Circulating Nucleic Acid extraction kit in accordance with the manufacturer’s standard instructions. The quantity and quality of all RNA extracts was determined using the QuBit fluorimeter (Invitrogen) and Agilent BioAnalyzer (with Small RNA chip) respectively. Small RNA sequencing libraries were preparing using the TruSeq Small RNA kit according to the manufacturer’s standard directions. Sequencing was conducted on the Illumina HiSeq 2000 platform with the aim of obtaining >5,000,000 reads per sample.

### Bioinformatic analysis

All sequencing data underwent stringent quality control measures prior to analysis. Briefly, reads were quality clipped to only retain reads with a median quality of ≥Q30, sequencing adapters removed, and read length distributions assessed to ensure these met the expectations of a miRNA sequencing run. An extract and count routine was utilised to condense the millions of sequencing reads into sequence tab count format to increase computational efficiency. Reads shorter than 15 nucleotides and longer than 35 nucleotides were filtered given that these were unlikely to map to miRNAs. Following parsing to remove superfluous data columns, the resulting tally tables comprising the miRNA sequence and count were processed by miRanalyzer V0.2 [6]. Reads were mapped to the human genome (hg18) and to miRBase version 20.0 permitting one mismatch between the sequencing reads and each index. Reads mapping to known miRNAs were counted and a relative expression value determined by dividing the number of reads mapping to each particular miRNA by the total number of reads mapped to all miRNAs. MicroRNAs evidenced by less than 10 sequencing reads or present in less than three study individuals were excluded prior to further analysis. Differential expression analysis and P value estimation were performed using QluCore Omics Explorer. The significance of differences between the various factors (BMI, smoking status and gender) was determined using a two-tailed t-test. In each case, contributions from other factors were eliminated using QluCore Omics explorer.

## Additional file

10.1186/s12867-016-0057-9 Sheet 1: Comprehensive list of raw and normalised expression values for all plasma miRNAs detected. **Raw Sequencing Data**—The raw sequencing data (in fastq format) supporting the results of this article is available in the Short Read Archive (SRA) repository, [SRP064104 http://www.ncbi.nlm.nih.gov/sra]. Sheet 2: Experiment IDs, PubMed IDs and metadata relating to the publically accessible datasets accessed for result validation.
